# Is a low level of free thyroxine in the maternal circulation associated with altered endothelial function in gestational diabetes?

**DOI:** 10.3389/fphar.2014.00136

**Published:** 2014-06-06

**Authors:** Enrique Guzmán-Gutiérrez, Carlos Veas, Andrea Leiva, Carlos Escudero, Luis Sobrevia

**Affiliations:** ^1^Group of Research and Innovation in Vascular Health, Facultad de Ciencias de la Salud, Universidad San SebastianConcepción, Chile; ^2^Facultad de Ciencias de la Salud, Universidad San SebastiánConcepción, Chile; ^3^Cellular and Molecular Physiology Laboratory, Division of Obstetrics and Gynaecology, School of Medicine, Faculty of Medicine, Pontificia Universidad Católica de ChileSantiago, Chile; ^4^Vascular Physiology Laboratory, Group of Investigation in Tumor Angiogenesis, Department of Basic Sciences, University of Bío-BíoChillán, Chile; ^5^University of Queensland Centre for Clinical Research, Faculty of Medicine and Biomedical Sciences, University of QueenslandHerston, QL, Australia

**Keywords:** thyroxine, gestational diabetes, endothelial dysfunction, pregnancy

## Abstract

Synthesis of thyroid hormones, thyroxine (T_4_) and tri-iodothyronine (T_3_), in the human fetus starts from 17 to 19th weeks of gestation. Despite the majority of normal pregnant women reaching adequate levels of circulating thyroid hormones, in some cases, women with normal pregnancies have low level of free T_4_ during first trimester of pregnancy, suggesting that T_4_ action may be compromised in those women and their fetuses. In addition, pathological low levels of thyroid hormones are detected in isolated maternal hypothyroxemia (IMH) and clinical hypothyroidism. Nevertheless, human placenta regulates T_3_/T_4_ concentration in the fetal circulation by modulating the expression and activity of both thyroid hormone transporters (THT) and deiodinases. Then, placenta can control the availability of T_3_/T_4_ in the feto-placental circulation, and therefore may generate an adaptive response in cases where the mother courses with low levels of T_4_. In addition, T_3_/T_4_ might control vascular response in the placenta, in particularly endothelial cells may induce the synthesis and release of vasodilators such as nitric oxide (NO) or vasoconstrictors such as endothelin-1 mediated by these hormones. On the other hand, low levels of T_4_ have been associated with increase in gestational diabetes (GD) markers. Since GD is associated with impaired placental vascular function characterized by increased NO synthesis in placental arteries and veins, as well as elevated placental angiogenesis, it is unknown whether reduced T_4_ level at the maternal circulation could result in an altered placental endothelial function during GD. In this review, we analyze available information regarding thyroid hormones and endothelial dysfunction in GD; and propose that low maternal levels of T_4_ observed in GD may be compensated by increased placental availability of T_3_/T_4_ via elevation in the activity of THT and/or reduction in deiodinases in the feto-placental circulation.

## INTRODUCTION

Thyroid gland produces tetra-iodothyronine (T_4_ or thyroxine) and tri-iodothyronine (T_3_). In the human fetus, the synthesis of these hormones starts from 17 to 19th weeks of gestation (wg), therefore it is well accepted that before this period, the circulating T_3_/T_4_ in the fetus depends on the maternal levels of these hormones (Pérez-López, 2007). Thus, an altered function of the thyroid gland at the maternal side could prejudice physiological levels of T_3_/T_4_ at the fetal circulation, and impair fetal growth and development. Worldwide studies indicate that ~10% of women may have hypothyroidism in their childbearing age (Mosso et al., 2012; Khalid et al., 2013; Ohashi et al., 2013). It has been also described that ~35 or ~3% of women with an apparent normal pregnancy have clinical hypothyroidism or exhibit maternal hypothyroxemia (low level of free T_4_), respectively (Mosso et al., 2012), both maternal conditions associated with several alterations in the fetus development (Parkes et al., 2012). Nevertheless, isolated maternal hypothyroxemia (IMH), a pathological condition manifested during pregnancy (Sahay and Nagesh, 2012), have been associated with occurrence of gestational diabetes (GD; Olivieri et al., 2000), pre-eclampsia (Sardana et al., 2009), or intrauterine growth restriction, IUGR; Chan et al., 2006). Moreover, pregnant women with IMH have higher risk (fourfold) to develop insulin resistance and GD (Karakosta et al., 2012; Tudela et al., 2012). In fact, reduced T_4_ level in the maternal circulation is associated with an increase in the incidence of GD pregnancies (Olivieri et al., 2000; Tudela et al., 2012) and with altered development of the central nervous system in children from pregnancies affected by these diseases (Smallridge and Ladenson, 2001; Casey et al., 2005). In addition, an incidence as high as ~70% of women coursing with pregnancies affected with GD exhibit IMH (Olivieri et al., 2000).

Gestational diabetes (GD) is associated with higher synthesis and release of vasodilators such as nitric oxide (NO) in the human fetal endothelium from GD (described as altered endothelial function) (De Vriese et al., 2000; Guzmán-Gutiérrez et al., 2011; Westermeier et al., 2011; Salomón et al., 2012). In addition, thyroid hormones are also involved in NO synthesis and release (Napoli et al., 2001; Fazio et al., 2004), but the potential contribution of reduced circulating T_4_ on deregulation of fetal endothelial function seen in GD pregnancies is unclear. We here analyze the available information regarding the potential relationship between maternal and fetal thyroid hormones with the occurrence of endothelial dysfunction in GD. We propose that the low maternal levels of T_4_ seen in GD may be compensated by higher placental availability of thyroid hormones via elevation in the activity of placental thyroid hormone transport and metabolism.

## OVERVIEW OF SYNTHESIS AND RELEASE OF THYROID HORMONES

The thyroid hormones 3,5,3′,5′-tetraiodothyronine (T_4_ or thyroxine) and 3,5,3′-triiodothyronine (T_3_) are synthesized in the thyroid gland and is regulated by hypothalamus/pituitary/thyroid axis by a negative feedback. In this regulatory axis, hypothalamus releases thyrotropin releasing hormone (TRH), which interacts with TRH receptors in thyrotropin cells in the pituitary gland to release thyroid stimulating hormone (TSH). In turn, TSH is the main regulator of the release of thyroid hormones leading to TSH receptor (TSHr) activation and increased iodo (iodide) uptake in the thyroid gland (Szkudlinski et al., 2002).

Iodide intracellular uptake is mediated by cotransport with sodium (Na^+^/I^-^) in the basal membrane of follicular cells in the thyroid gland. In these cells, iodides are oxidized by thyroid peroxidase (TPO) in the presence of hydrogen peroxide. Iodine (oxidized iodine) binds to thyroxine residues belong to the tiroglobulins (Tg), then tyroxine residues can be mono (MIT), di (DIT), tri (T_3_), or tetra-iodinated (T_4_) (Sugenoya et al., 1984; Rousset, 1991). The release of thyroid hormones through the basolateral membrane in thyroid gland follicular cells requires endocytosis of iodinated Tg at the apical side of these cells. The Tg is then incorporated into phagolysosomes and digested by proteolytic proteins, with MIT and DIT being re-uptaked into Tg; however, T3 and T4 are released toward circulation (Rousset, 1991). T_4_ is the main thyroid hormone released by thyroid gland follicular cells (~40-fold compared with T_3_) and is almost all (99.97%) bound to thyroxine binding globulin (TBG), albumin and pre-albumin in the circulation. In addition, free T_3_ accounts for ~0.3% and the rest is bound to TBG and albumin.

Free T_3_ is the hormone with biological activity and is the active form of thyroid hormones. It is derived from 5′-deiodination of free T_4_ via iodothyronine deiodinases located in the target tissues (Sugenoya et al., 1984; Rousset, 1991; Schussler, 2000; Bianco and Kim, 2006). Deiodinases are grouped in three subtypes: I, II, and III (or D1, D2, and D3, respectively), all of which are involved in the regulation of T_3_ activity (Bianco and Kim, 2006; Darras and Van Herck, 2012). For instance, D2 is specific to generate T_3_ from T_4_; however, D3 generates DIT from T_3_, and reverse T_3_ (rT_3_, inactive form of T_3_) from T_4_ (Kilby et al., 2005). D1 has been reported as an enzyme that is much less active compared with the other forms (Bianco and Kim, 2006; Dentice and Salvatore, 2011; Darras and Van Herck, 2012).

## VASCULAR EFFECTS OF THYROID HORMONES

Thyroid hormones reduce peripheral vascular resistance by promoting relaxation in human and murine vascular smooth-muscle cells (Klemperer et al., 1995; Ojamaa et al., 1996; Park et al., 1997), and improve vascular reactivity by endothelium-dependent and -independent mechanisms (Napoli et al., 2001; Fazio et al., 2004). In rat, T_4_ released from the mesenteric arteries increases vasorelaxation when administrated at supraphysiological concentrations (Zwaveling et al., 1997). Moreover, T_3_ and T_4_ improve fibroblast growth factor-2 (FGF-2) expression, a recognized proangiogenic factor, in cultures of ECV304 cells (Davis et al., 2004). Interestingly, human umbilical vein endothelial cells (HUVEC) exposed to high T_3_ levels exhibit high expression of endothelin-1 (vasoconstrictor) and fibronectin (profibrotic molecule), suggesting that pathological conditions such as hyperthyroidism could be associated with vasoconstriction (Baumgartner-Parzer et al., 1997; Diekman et al., 2001).

**FIGURE 1:**
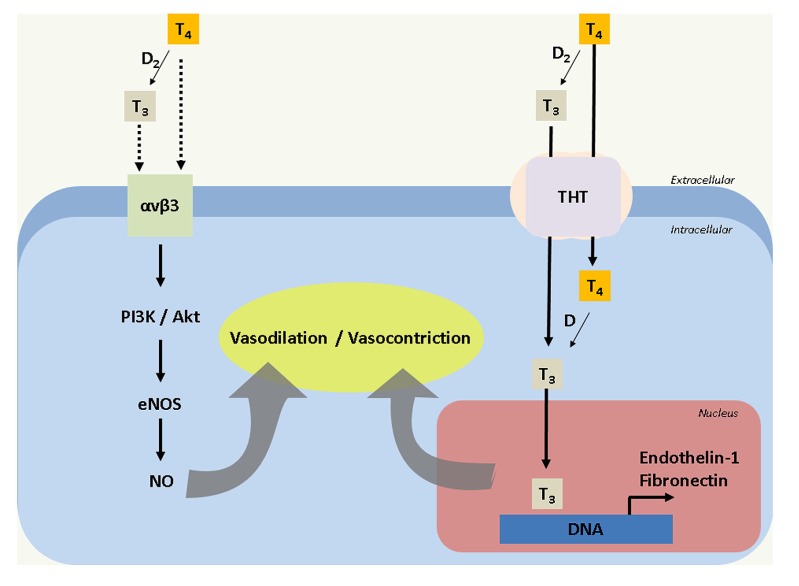
**Thyroids hormones effects on endothelial cells**. T_4_ (Tyrosine) is metabolized by deiodinase 2 (D_2_) to T_3_ (triiodothyronine) of manner extra and intracellular. T_3_ and T_4_ (Tyrosine) could activates αvβ3 integrin receptor which initiate a signaling that involve PI3K, Akt and eNOS, generating nitric oxide (NO) as vasodilator. But, also T3 and T4 are uptake by thyroid hormones transporters (THT). T3 bind to DNA and activate transcriptional activity for endothelin-1 and fibronectin which are associated with vasoconstriction.

In addition, there is evidence that vasoconstriction associated with high levels of thyroid hormones may result from a non-genomic action, which seems mediated by αvβ3 integrin as reported in HUVEC (Luidens et al., 2010). In this regard, αvβ3 integrin is a membrane protein that should active the phosphatidylinositol 3 kinase and protein kinase B/Akt (PI3K/Akt) pathway in this cell type (Hiroi et al., 2006; Luidens et al., 2010). Nevertheless, in another study HUVEC and bovine aortic endothelial cells (BAEC) seems to respond to T_3_ by increasing the phosphorylation of serine^1177^ (Ser^1177^; Ser^1177^-eNOS) at the endothelial nitric oxide synthase isotype (eNOS) in a time- and concentration-dependent manner (Hiroi et al., 2006). Similar results were seen in vascular smooth-muscle cells from rat thoracic aortae where T_3_ increases Ser^1177^-eNOS via PI3K/Akt pathway inducing eNOS, inducible (iNOS) and neuronal (nNOS) NOS expression. Thus, it is likely that T_3_ increases NOS expression via a genomic and a non-genomic (i.e., via αvβ3 integrin) action (Carrillo-Sepúlveda et al., 2010). Therefore, the thyroid hormone concentration is a determinant factor involved in the modulation of vascular function (**Figure 1**). However, there is not information addressing thyroid hormone effects on the human feto-placental vasculature.

## HUMAN THYROID HORMONES IN PREGNANCY

Human fetal thyroid hormones are secreted from the 17 to 19 wg, indicating that the fetus requires thyroid hormones delivery from the mother during the first and beginning of the second trimester of pregnancy (Pérez-López, 2007). Pregnant women have TSH and free T_4_ levels that are normal and comparable to those in non-pregnant women; however, in the first trimester of pregnancy, there is an increase in the maternal free T_4_ level most likely in response to chorionic gonadotropin hormone (hCG; Fantz et al., 1999; Pérez-López, 2007). It has been shown that increased hCG level leads to reduced hypothalamus/pituitary/thyroid axis activity, but improved thyroid hormones delivery to the fetus in the first trimester of pregnancy (Fantz et al., 1999; Pérez-López, 2007; Chan et al., 2009). In this period, the concentration of free T_4_ in the fetal circulation corresponds to a third of the level found in the maternal circulation (Chan et al., 2009). This phenomenon results from a reduced concentration of the TBG binding protein in the fetal circulation, which leads to a free T_4_ concentration enough to exert its biological effects in embryonic tissues (Kilby et al., 2005; Chan et al., 2009). In the second trimester of pregnancy, free T_4_ levels in the fetal circulation corresponds to about half of the concentration detected in the maternal circulation (Kilby et al., 2005; Chan et al., 2009). Therefore, it seems clear that thyroid hormone levels are regulated by the placenta tissue (Burrow et al., 1994). Thus, a role of this organ is crucial in the delivery of T_4_ to the fetus.

## THYROID HORMONES METABOLISM IN THE HUMAN PLACENTA

In addition to the paracrine effect of hCG on the hypothalamus/pituitary/thyroid axis, the human placenta regulates directly the thyroid hormone concentration in the fetal circulation by modulation of thyroid hormone transporters (THT), and by thyroid hormones metabolism mediated by deiodinases (Burrow et al., 1994). THT are located at the apical and basolateral membranes of the cytotrophoblasts, syncytiotrophoblast and microvascular endothelial cells (James et al., 2007). There are several THT, including monocarboxylate transporters (MCT), where MCT8 and MCT10 are the main forms. Moreover, a role has been reported for L-amino acid transporters (LAT) and organic anion transporter polypeptides system (OATPs), which operates with less selectivity for T_4_ (James et al., 2007). Also in the human placenta MCT8 (Park and Chatterjee, 2005), MCT10, LAT1, LAT2 (Friesema et al., 2003), OATP1A2 and OATP4A1 (Hagenbuch and Meier, 2003; Hagenbuch, 2007) have been identified, but no studies addressing the role of these membrane transporters in any pathology of pregnancy have been documented. Moreover, D2 (located at the endoplasmatic reticulum) and D3 (located at the plasma membrane with a cytoplasmic active site; Koopdonk-Kool et al., 1996; Stulp et al., 1998; Chan et al., 2003) have been identified. D2 and D3 are referred as major factors controlling transplacental transport of T_4_ to the fetus (Mortimer et al., 1996). Interestingly, D2 and D3 expression is up regulated by T_3_ (Chan et al., 2003). In addition, since changes in the level of T_3_/T_4_ cause altered THT expression (Mortimer et al., 1996), modulation of D2/D3 and THT expression by T_3_/T_4_ could be a phenomenon serving as a defense mechanism for the fetus in pregnancies where the mother courses with hypothyroxemia.

## ISOLATED MATERNAL HYPOTHYROXEMIA AND CLINICAL HYPOTHYROIDISM

The pathologies associated with low levels of free T_4_ correspond to IMH and clinical hypothyroidism. IMH (1–2% of normal pregnancies) is characterized by low free T_4_ (<10th percentile in normal range), but normal TSH level. Instead, clinical hypothyroidism characterizes by high levels of TSH, but low levels of free T_4_ (Casey et al., 2005). In another pathological condition referred as “low T_3_ syndrome,” an increase in the expression of the membrane transporters MCT8 is reported, which could be a compensatory response to low levels of thyroid hormones (Mebis et al., 2009). The latter seems paralleled by an increase in the D1 and D2 levels as reported in human skeletal muscle and liver (Peeters et al., 2003; Weetman et al., 2003). While, using a *knockout* mice model for MCT8 (*Mct8*^-/-^) an increase in the plasma free T_4_ and T_3_ levels, and D1 and D2 expression and activity in the liver was shown (Dumitrescu et al., 2006). Therefore, these results support the fact that low levels of T_4_ lead to changes in the THT and deiodinase expression and activity in target organs. However, there is no information addressing this possibility in the human placenta from pregnancies coursing with maternal hypothyroxemia.

On the other hand, minor changes such as D2 gene polymorphism (Thr92Ala) are associated with human type 2 diabetes mellitus and insulin resistance (Mentuccia et al., 2002). In this regard, despite there is not information regarding D2 gene polymorphism in women coursing with pregnancies without a diagnosis of thyroid gland pathology, a negative correlation between free T_4_ level and metabolic markers of GD and insulin resistance (i.e., degree of glycosylated HbA_1c_, fasting insulin, and HOMA-IR) has been shown (Bassols et al., 2011). Then, it is proposed that a potential relationship between low maternal free T_4_ levels and occurrence of GD and perhaps its complications including endothelial dysfunction exists.

## MATERNAL T_4_ LEVEL AND GD

GD is a disease coursing with glucose intolerance first recognized or manifested during pregnancy [Metzger et al., 2007; Reece et al., 2009; American Diabetes Association [ADA], 2012]. This pathology accounts for ~5% of pregnant women worldwide and it is associated with high risk of fetal perinatal alterations (e.g., macrosomia, insulin resistance) and higher incidence of diseases in the adulthood (e.g., GD, obesity, dyslipidemia, hypertension, metabolic syndrome) (Poston, 2010; Negrato et al., 2012). GD is associated with reduced maternal circulating T_4_ levels in the first trimester of pregnancy. To date, 5% of women coursing with GD pregnancies have been shown to correlate with IMH (Krcma et al., 2010). In addition, free T_4_ levels are lower in women with GD pregnancy compared with women with normal pregnancies (Velkoska-Nakova et al., 2010) and a reduced free T_4_ level is shown in 70% of patients with GD pregnancies (Olivieri et al., 2000). Therefore, maternal hypothyroxemia could be associated with GD.

Other pathologies associated with low levels of free T_4_, such as clinical hypothyroidism (i.e., low free T_4_ and high TSH levels), also have been associated with GD. Indeed, 6–15% of GD pregnancies are associated with hypothyroidism (Velkoska-Nakova et al., 2010; Tudela et al., 2012; Stohl et al., 2013). Moreover, if pregnant women have hypothyroidism, they have 4.3-fold higher risk for developing GD (Karakosta et al., 2012). There are no publications addressing the T_4_ plasma levels at the fetal circulation in a pregnancy coursing with GD. However, since GD courses with endothelial dysfunction (De Vriese et al., 2000; Westermeier et al., 2011; Guzmán-Gutiérrez et al., 2011, 2014; Salomón et al., 2012) and thyroid hormones modulate endothelial function (Napoli et al., 2001; Fazio et al., 2004), it is likely that a low free T_4_ level at the maternal circulation eventually could result in altered endothelial function in GD pregnancies.

## GD AND ENDOTHELIAL DYSFUNCTION

One of the main alterations detected in GD pregnancies is the associated endothelial dysfunction of the fetoplacental circulation (De Vriese et al., 2000; Guzmán-Gutiérrez et al., 2011, 2014; Westermeier et al., 2011; Salomón et al., 2012). Since the vasculature in the human placenta lacks innervation (Marzioni et al., 2004), several local metabolic mechanisms, such as synthesis and release of vasoactive molecules (e.g., NO, adenosine) (Vásquez et al., 2004; Guzmán-Gutiérrez et al., 2011, 2014; Sobrevia et al., 2011) or release of nanovesicles (e.g., exosomes), most likely mediating autocrine and/or paracrine modulation of vasculature (Salomon et al., 2013), could lead to acute and rapid modulation of vascular tone in this vascular bed (Guzmán-Gutiérrez et al., 2011, 2014; Burnstock and Novak, 2013).

Arteries and veins in the human placenta from pregnancies with GD exhibit increased NO synthesis (Figueroa et al., 2000). Furthermore, similar results were early reported in primary cultures of HUVEC from pregnant women diagnosed with GD (Sobrevia et al., 1995). Therefore, it has been proposed that vascular dysfunction in GD could result from a functional dissociation between NO synthesis and its bioavailability in the human placental circulation (Guzmán-Gutiérrez et al., 2011, 2014; Sobrevia et al., 2011). Even when endothelial dysfunction, referred to as an alteration of NO synthesis and the uptake of cationic amino acid L-arginine (i.e., L-arginine/NO pathway), is associated with GD, a clear mechanism behind these effects of GD is still unavailable (Guzmán-Gutiérrez et al., 2014).

## HUMAN PLACENTA ENDOTHELIAL FUNCTION AND T4 IN GD: A HYPOTHESIS TO BE TESTED

**FIGURE 2:**
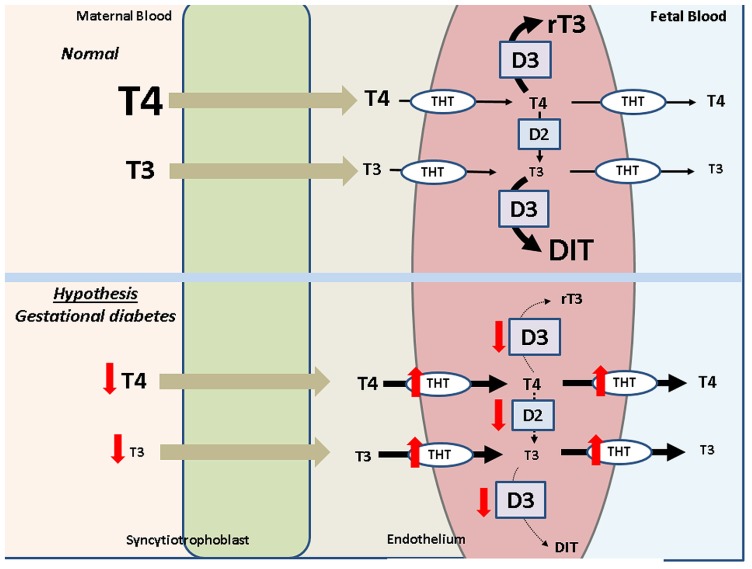
**Potential thyroid hormones transplacental transport in gestational diabetes**. In cells from normal pregnancies (*Normal*) T3 and T4 in the maternal blood are transported to the fetal blood by thyroid hormone transporters (THT) through the microvascular endothelium (*Endothelium*). T3 and T4 are metabolized by deiodinases 2 (D2, forming T3) and 3 (D3, forming reverse T3 9 [T3 from T4 and diiodothyronine (DIT) from T3)], thus leading to modulation of these hormones delivery to the fetal blood. As hypothesis, in *Gestational diabetes* the mother courses with reduced T3 and T4 levels (red arrows). There is an increase THT and reduced D2/D3 activity, thus compensating T4 level in the fetal blood.

In normal pregnancies maternal free T_4_ is taken up by THT by the syncytiotrophoblast, where it is metabolized by D2 and D3 to be converted into T_3_ or rT_3_, respectively. T_4_ is then released via THT at the basolateral membrane of the syncytiotrophoblast into the intervillous space from where it is taken up by the microvascular endothelial cells via THT. In these cells a fraction of T_4_ is metabolized again to produce T_3_ and rT_3_ via D2 and D3, respectively (**Figure 2**). As a result of this process, T_4_ and T_3_ are released into the fetal blood. However, currently there is no information regarding transport of thyroid hormones across the human placenta in GD pregnancies. We propose that low levels of T_4_, lead to an increase in the number and activity of THT membrane transporters available at the plasma membrane of the human placental endothelial cells, and reduced deiodinase expression and activity, in order to supply T_4_ necessities associated with fetal development in GD. These changes could constitute a mechanism by which the endothelium from the human placenta intends to maintain normal intracellular and circulating levels of T_4_ in the fetus. The latter would be potentially reached by a greater delivery of maternal T_4_ to the fetal blood.

## FINAL COMMENTS AND CONCLUSION

Based on what was described in this review, our central research questions are: (1) is a low level of free T_4_ in the maternal circulation associated with GD? (2) is GD a disease associated with increased placental THT, but reduced deiodinase expression and activity? and (3) would the potential changes caused by reduced free T_4_ level in the maternal circulation and altered THT and deiodinases in the placenta in GD lead to placental endothelial dysfunction? Furthermore, nothing is known regarding the feto-placental vascular function/dysfunction in pregnancies where the mother courses with hypothyroxemia. Despite many benefits for using human placental tissue after birth, we acknowledge that information behind cellular mechanisms and adaptative response occurred at the beginning of pregnancy is difficult to extrapolate; however, it offers a good approximation for studying consequences of human pathologies. Potential more complex models, might include analysis of placentas collected from animal deficient in leptin receptor (*db*/*+*), since they develop GD during pregnancy (Bobadilla et al., 2010), offering a model that may to understand molecular mechanisms of THT and deiodinases in first trimester of pregnancy. Moreover, a therapeutical approach of pregnant women coursing with hypothyroxemia targeted to improve free T_4_ circulating levels will likely reduce the risk of developing GD and the deleterious consequences of this disease in the feto-placental endothelial function. We also speculate that a normalization of free T_4_ levels in the first trimester of pregnancy could reduce the risk to face GD-associated complication.

## AUTHOR CONTRIBUTIONS

Enrique Guzmán-Gutiérrez and Luis Sobrevia generated the text and figures, Carlos Veas, Andrea Leiva, and Carlos Escudero contributed for design of text.

## Conflict of Interest Statement

The authors declare that the research was conducted in the absence of any commercial or financial relationships that could be construed as a potential conflict of interest.
